# A Case of Toxic Epidermal Necrolysis Caused by Lamotrigine Combined With Valproic Acid and Literature Review

**DOI:** 10.7759/cureus.45334

**Published:** 2023-09-16

**Authors:** Haifeng Liu, Jia Yang, Ruoyang Liu

**Affiliations:** 1 Center for Rehabilitation Medicine, Run Shaw Hospital Branch, Zhejiang University School of Medicine, Zhoushan, CHN

**Keywords:** psychiatric symptoms, toxic epidermal necrolysis, adverse drug reactions, sodium valproate, lamotrigine

## Abstract

After suffering from frequent symptomatic seizures secondary to cerebral hemorrhage, a 58-year-old male patient was prescribed a one-time 50 mg dose of lamotrigine, which he took for a week. However, the patient's seizure symptoms were not controlled until a dosage of 500 mg of sodium valproate tablets was taken twice daily, which ultimately resolved his seizures. Unfortunately, about two weeks after the combination, the patient developed a rash. Nine days later, the patient developed new blisters, necrotizing epidermal desquamation, and lesions over 80% of their body surface area. This was diagnosed as toxic epidermal necrolysis (TEN) resulting from the combination of lamotrigine and sodium valproate. The sodium valproate and lamotrigine were discontinued and treated symptomatically for about one month. The patient's condition improved as the fatal rash gradually subsided. However, after the onset of TEN, unexpected psychiatric symptoms such as poor sleep, less than four hours of sleep, irritability, paranoia, crying, fear of rash recurrence, and suspicious hallucinations and delusions emerged in the patient. Surprisingly, after discontinuation of lamotrigine and sodium valproate due to the rash, the patient did not experience any further seizures.

## Introduction

Toxic epidermal necrolysis (TEN) is the most common adverse effect of medications, such as antibiotics, nonsteroidal anti-inflammatory drugs, and allopurinol. This disease is known for its high mortality rate, often resulting in skin metaplasia. While lamotrigine is generally a well-tolerated anticonvulsant drug, it can still trigger Stevens-Johnson syndrome (SJS) and TEN, albeit rarely. The incidence rate of these conditions is approximately 1:1,000 in adults and 3:1,000 in children [1]. In the clinical management of epilepsy, combining lamotrigine and valproate has been shown to yield better therapeutic results [2]. However, it is important to note that the risk of developing SJS or TEN increases significantly when these two medications are used together, compared to when they are used alone [3]. Additionally, the occurrence of severe rashes caused by multiple drugs is often overlooked and should not be ignored. In cases like lamotrigine and valproic acid (VPA), it is often difficult to accurately identify the onset of SJS or TEN. Additionally, the current treatment approach is not very comprehensive, and there are certain symptoms that remain due to the rash, including its prognosis. We have written a report that details the changes in the patient's rash throughout the onset of TEN and documents the complete treatment process. Our aim is to provide a reference for the safe use of medication in the treatment of symptomatic epilepsy secondary to cerebral hemorrhage.

## Case presentation

The patient, a 58-year-old male from China, was admitted to the hospital for treatment of a rash that appeared two days ago (namely February 2, 2022). A review of the patient's history prior to this admission: On January 25, 2020, the patient experienced a right basal ganglia hemorrhage following a sudden increase in blood pressure (approximately 200 mmHg/100 mmHg). It is important to note that the patient did not experience any significant sequela after medical treatment and did not report any occurrence of epileptic symptoms. Upon discharge from the hospital, the patient did not adhere to the prescribed antihypertensive medication regimen or regularly measure their blood pressure. Unfortunately, on August 7, 2021, the patient suffered another cerebral hemorrhage in the right basal ganglia due to a sudden increase in blood pressure. This time, the hemorrhage was more severe, leading to the patient undergoing a “craniotomy for removal of intracranial hematoma and decompression with debridement flap” procedure. After that, the patient was suffering from residual dyskinesia of the left upper and left lower limbs. As a result, the patient intermittently came to our rehabilitation department for rehabilitation training. However, during the rehabilitation training period, the patient experienced frequent limb convulsions on the left lower limb, leading to a diagnosis of symptomatic epilepsy secondary to cerebral hemorrhage through EEG on January 2, 2022. The patient was initially prescribed oral lamotrigine (LTG) at a dosage of 50 mg once per day. After one week, the dosage was increased to 50 mg twice per day, but after about another week, the patient continued to experience frequent convulsions. VPA extended-release tablets were then added to the patient's regimen at a dosage of 500 mg twice per day, which effectively controlled their seizure symptoms. The patient was discharged from the hospital with regular oral medication as previously described. The patient consumed some seafood, and about two weeks after the combination, his skin rapidly turned red without any itching, pain, or ulceration. Because of this rash, the patient was readmitted to our hospital.

**Figure 1:**
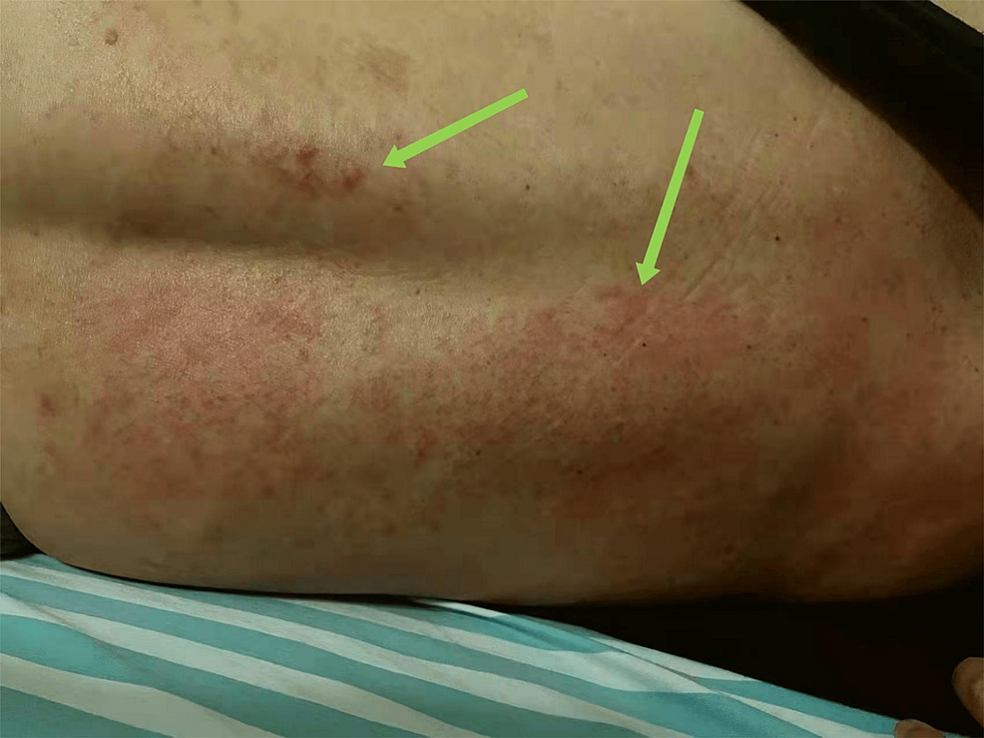
The skin of back had a reddish-colored maculopapular rash, partially fused into small flakes on February 4, 2022 This image depicts the patient's back two days after the rash appeared. The arrows highlight a reddish-colored maculopapular rash on the patient's back, resembling scattered soybean grains that partially fused into small flakes.

His past medical history includes hypertension not regularly treated with medication (amlodipine benzenesulfonate), and he takes calcium carbonate D3 tablets year-round unless he takes LTG and VPA. The patient reported no history of drug or seafood allergies and had no notable family history or prior history of smoking or drinking. During admission, the patient had a temperature of 38℃, a pulse rate of 80 times/min, a respiratory rate of 16 times/min, and a blood pressure of 127/82 mmHg. The physical examination revealed that the patient was conscious but physically weak. The patient's skin had a reddish-colored maculopapular rash, resembling scattered soybean grains that partially fused into small flakes, as depicted in Figure 1. During the examination, the focus was on the chest and back of the body, where no pain or ulcerated skin was observed upon touch. The muscle strength of the left upper limb was graded as 2, while the muscle strength of the left lower limb was graded as 3. On the right side, the muscle strength was graded as 5, and muscle tone was normal. No other abnormalities were observed during the examination. The patient was admitted to the hospital for routine blood, coagulation, biochemistry, plasma-D dimer, urine, and stool tests. The results showed an eosinophil count of 0.56×10^9^/L (reference value 0.05-0.52×10^9^/L), serum amyloid of 17.63 mg/L (reference value 0-10 mg/L), and glucose level of 6.12 mmol/L. No other significant abnormalities were found. The admission diagnosis includes investigation of a rash, symptomatic epilepsy secondary to cerebral hemorrhage, recovery from cerebral hemorrhage, left hemiparesis, and high blood pressure. After consultation with a dermatologist, he said that it could not be ruled out that the allergic rash was caused by food or drugs, and he agreed to stop using lamotrigine. For anti-allergic treatment, a 10 mg endovenous infusion of dexamethasone sodium phosphate injection, a 10 mg intramuscular injection of chlorpheniramine maleate, a 5 mg levo cetirizine hydrochloride tablet, and a 5 mg loratadine tablet were administered once a day. In addition, a 40mg intravenous infusion of omeprazole injection was given to prevent stress ulcers.

**Figure 2:**
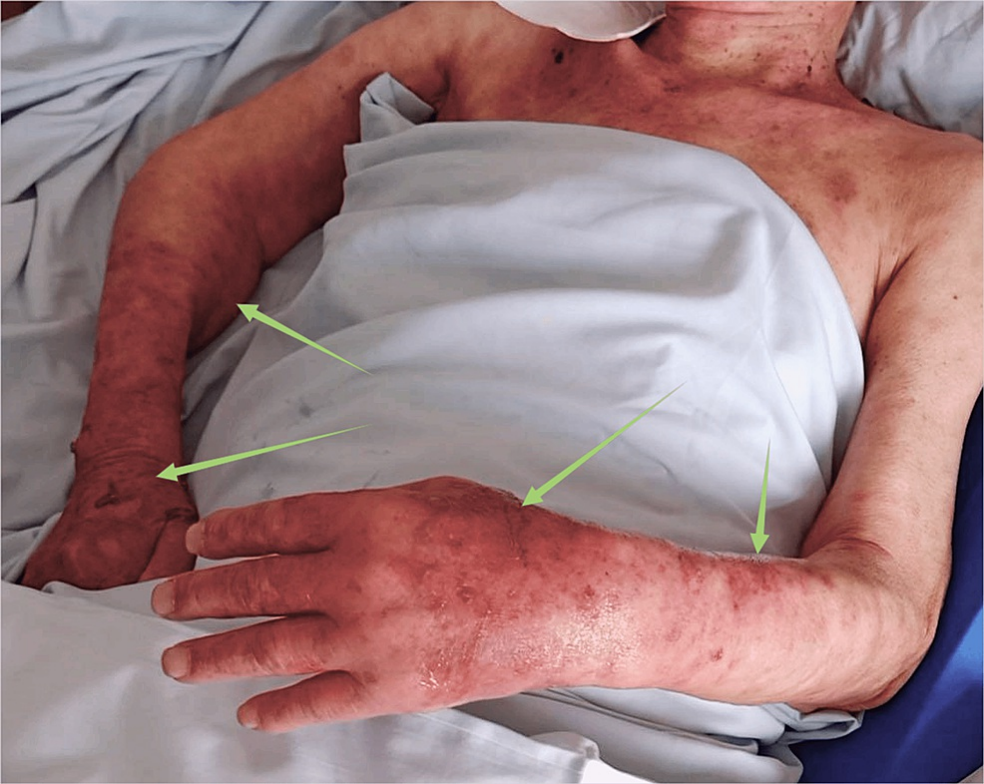
Blood-red fine papules on both upper extremities of the patient on February 6, 2022 The arrows show small, bright, blood-red maculopapular eruptions on the patient's inner sides of both upper limbs.

On February 6, 2022 (four days after the onset), the patient's temperature was recorded at 36.9°C, with a pulse rate of 69 times/min, respiration rate of 16 times/min, and blood pressure of 126/78 mmHg. The patient reported experiencing skin tenderness at the rash site, which was tolerable. During the physical examination, it was observed that the initial rash had progressed to a darker shade of red and had a tendency to merge into patches. Additionally, the rash on the inner parts of the limbs also increased in size and spread outwards. The patient developed small, bright, and blood-red maculopapules on the inner sides of both upper limbs, as depicted in Figure 2. As the rash progressed, the patient discontinued dexamethasone and started receiving immune suppression treatment with normal saline 100 mL, methylprednisolone sodium succinate 80 mg intravenous infusion previously day. Additionally, the patient received a 5% glucose sodium chloride injection of 500 mL and a multivitamin injection of 5 mL endovenous drip at one time daily along with vitamin supplements.

On February 8, 2022 (six days after the onset), the patient's temperature was recorded at 37.1°C, pulse at 80/min, respiration at 16/min, and blood pressure at 130/76 mmHg. The patient reported significant tenderness at the skin lesions, particularly at night. During examination, a dark-red rash on the back of the chest was observed, which appeared to be fused into a patch. The rash also seemed to have an intercalated layer containing clear mucus, which did not ulcerate. The patient presented with yellow bean-sized blisters on the internal side of both upper extremities filled with evident fluid that did not ulcerate. The rash on the interior side of both lower extremities was red and unilaminate. Additionally, there were bright-red macules on the dorsal side of the right foot and dark purple petechiae between the caudal sacrum and the buttock. The patient's blood test results showed a WBC count of 11.8×10^9^/L and an eosinophil count of 1.66×10^9^/L. The serum amyloid level was 45.29 mg/L, while the direct bilirubin level was 1.5 µmol/L (reference value 1.7-6.8 µmol/L), and the total protein level was 58.8 g/L (reference value 65-85 g/L). The ALT level was 84 IU/L (reference values 9-60 IU/L). Due to the worsening of the patient's rash, there was a risk of serious infection if it broke down. Therefore, it was recommended that the patient be transferred to a special burn unit. However, the patient's family refused. The possibility of a rash could not be eliminated. Prior to admission, the patient took oral medicine, resulting in the discontinuation of sodium valproate and calcium carbonate D3 tablets. To prevent infection, 100 mL of normal saline and 2 g of cefoperazone sulbactam were administered intravenously once daily. Additionally, Hugan tablets, a traditional Chinese medicine that can effectively protect the liver and lower ALT, were taken orally three times a day. To improve skin care, it is important to pay attention to hand hygiene and ensure that medical staff wear masks and disposable sterilized gloves. Supplementally, family members and accompanying staff should be required to wear disinfected gloves when touching patients. It is also important to keep hospital beds dry and maintain a suitable room temperature to prevent possible ulceration and infection.

On February 11, 2022 (nine days after the onset), the patient's body temperature was 36.2°C, pulse was 74 beats/min, respiration was 16 breaths/min, and blood pressure was 125/95 mmHg. The patient experienced strong tenderness, was unable to lie down, and felt thirsty. During the physical examination, the patient's head and face were swollen, and the mucous membrane of the eyelids showed obvious congestion. The rash on the chest had fused together and was incompletely broken. The rash on the back appeared like a sandwich and had been partially cankerous with white plus exuding. The skin around the ulcerated area was exposed and looked similar to a scalded mucosa. There were also ulcers on the buttocks under pressure, which were oozing. Additionally, herpetiform changes were observed in the maxillae and sacrococcygeal area. The blisters on both upper limbs remained unchanged, but the surrounding rash turned dark red and fused into small patches. The rash on both lower limbs slightly increased without ulceration. There was also slight ulceration of the scrotum, penis, and anus. The patient presented with lesions covering more than 80% of their body. After consulting with a dermatologist, it was determined that blisters and bullae had developed in the armpit and sacrum of the coccyx. The presence of scattered bullae in the sacrum of the coccyx and trunk in the armpit, along with a positive Nissl sign, led to a diagnosis of TEN. White blood cell count was 9.1×10^9^/L, eosinophil count was 0.00×10^9^/L, serum amyloid was 84.65 mg/L, ALT was 68 IU/L, total protein was 55.3 g/L, albumin was 33.8 g/L, urine routine: white blood cells 1+(70) cacells/µL. The patient's rash progressed significantly, and he became immunocompromised, but his liver function was stable and improved. To improve skin dressing change care, it is recommended to disinfect the entire body before externally applying moist burn ointment twice a day.

**Figure 3:**
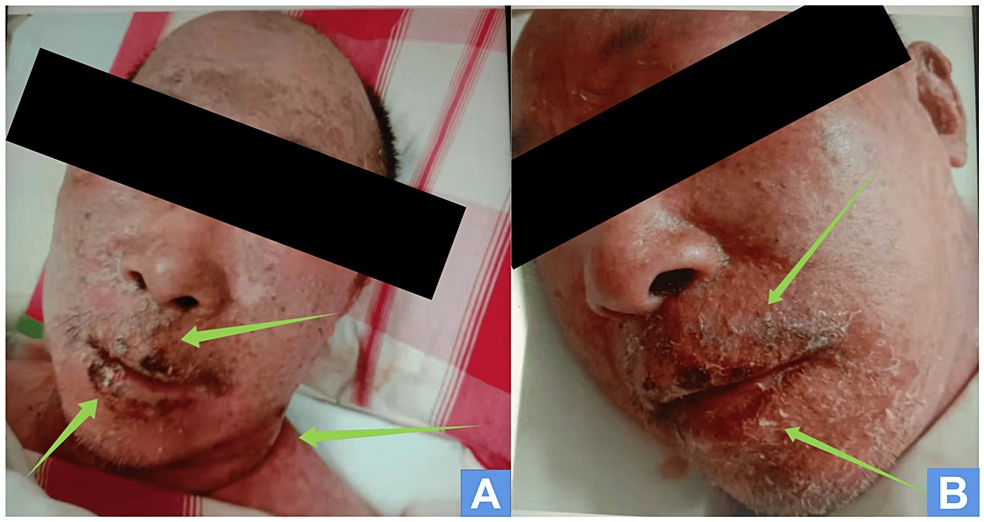
The patient sufferers with ocular complications on February 15, 2022 (A) Front face and neck, (B) Side face. Arrows show that the patient presented with mucosal ulceration and bleeding at the corners of the mouth, as well as incomplete rupture of the skin at the neck folds.

On February 15, 2022 (13 days after the onset), the patient presented with a temperature of 36.5°C, pulse rate of 66 times/min, respiratory rate of 16 times/min, and blood pressure of 122/74 mmHg. The patient also reported severe tenderness and thirst. During the physical examination, it was observed that the blisters had a tendency to increase in size. Additionally, there were scattered oral mucosal ulcers that were the size of rice grains. The eyelids had a large amount of secretions that had adhered to the skin surface surrounding the ruptured blisters. The patient presented with mucosal ulceration and bleeding at the corners of the mouth, as well as incomplete rupture of the skin at the neck folds, as depicted in Figures 3A, 3B. There was exposed adhesion of the epidermis and a dark-red rash on the chest that developed into large blisters, which were ulcerated and adhered. The exposed skin at the ulcers resembled shallow second-degree burns, with obvious exudation. The rash extended to the neck, shoulders, back, and waist, and fused into a large dark-red area with severe ulceration. The area where the ulcer was located had exposed the basal layer and was mixed with transparent exudate and dark-brown dead skin. Although most of the rash on the extremities had been ulcerated, no new rash was visible. To prevent potential Clostridium tetani infection due to the large-scale ulceration of the blisters and exposure through the skin, a temporary intramuscular injection of 1,500iu tetanus antidote was administered. For passive immunotherapy, administer 15 g of gamma globulin intravenously once daily and maintain it for three days. The patient experienced minor hemorrhaging in both eyes' conjunctiva and slight edema in the right temporal conjunctival layer of the bulb. Upon consulting an ophthalmologist, the patient was diagnosed with bulbar conjunctiva edema and conjunctivitis in the affected eye. The patient was treated with azelastine and levofloxacin eye drops, administered three times daily for seven days, along with Sodium acid eye water, applied four times daily for one month. The patient's dead skin was not completely removed to prevent further infection. Instead, the wound was washed with normal saline and treated with moist burned ointment. The whole body was covered with a non-adhesive medical gauze dressing.

On February 21, 2022 (19 days after the onset), the patient's body temperature was 36.8°C, pulse rate was 77 beats per minute, respiration rate was 16 breaths per minute, and blood pressure was 158/86 mmHg. The patient reported relief from pain. During the physical examination, a significant amount of affected skin was observed to be flaking off. Over time, the fluid oozing from the open sores on the torso and limbs decreased and eventually dried up, causing a significant amount of dead skin to flake off. The swelling in the head and face subsided, and a large amount of necrotic scalps were shed. The dark-brown dead skin on the face was also completely shed, revealing new, moist skin underneath. Additionally, the amount of fluid seeping from the damaged mucous membranes in the mouth and eyelids decreased. The affected skin showed signs of improvement, with red and white folds and shedding of dark-brown dead skin. The scrotum has also returned to almost normal size after significant retraction. Additionally, the mucosa in the oral cavity, corners of the mouth, and eyelids have improved, with decreased exudation from the trauma. The patient's oral cavity, corners of the mouth, and eyelids showed improvement in the damaged mucosa, and exudation from the trauma decreased. The patient's WBC count was 7.4×10^9^/L, with an eosinophil count of 0.00×10^9^/L. Additionally, the patient's serum amyloid was 19.53 mg/L, ALT was 57 IU/L, total protein was 58 g/L, albumin was 25.1 g/L, and glucose was 7.46 mmol/L. The patient's rash was controlled, and their condition improved. The patient's treatment plan included a switch to a 40mg micro-pump of Methylprednisolone sodium succinate, administered twice daily, and a daily intravenous dose of 10g human albumin for three days. Additionally, the patient was advised to consume high-protein foods such as whey protein powder, lean meat, eggs, and milk, and to supplement their diet with fruits and vegetables to optimize nutrition and vitamin intake.

On February 27, 2022 (25 days after the onset), the patient presented with a temperature of 37.1°C, a pulse rate of 71 beats per minute, a respiratory rate of 16 breaths per minute, and a blood pressure of 114/80 mmHg. The patient's pain significantly decreased. However, He began experiencing psychiatric symptoms, which belong to the symptomatic psychosis secondary to TEN, such as poor sleep, less than four hours of sleep, irritability, paranoia, crying, fear of rash recurrence, and suspicious hallucinations and delusions. During the examination, the patient reported seeing deceased patients multiple times throughout the day exhibited nonsensical speech, and expressing suspicions of infidelity by his wife after she had a brief conversation with other men. However, the rash on the patient's entire body had significantly subsided, and a large amount of dark-brown dead skin had fallen off. The broken epidermis dried and crusted, with new skin appearing primarily on the chest and back. The skin on the sacrococcygeal and buttocks area appeared dark red. In contrast, the inflammation of the eyelids, mucous membranes of the corners of the mouth, and oral ulcers have disappeared, and the scrotum has returned to its normal size. The hormone dosage was reduced to once a day. To aid in anti-anxiety and sleep, estazolam tablet 1 mg was administered orally before bedtime.

On March 3, 2022 (29 days after the onset), the patient's vital signs were as follows: temperature of 36.6°C, pulse rate of 61 beats/min, respiration of 17 breaths/min, and blood pressure of 121/72 mmHg. The patient's psychiatric symptoms remained unchanged from before, while the rash had mostly healed, leaving behind a few light black pigmentation spots on the inner extremities. The patient's intravenous corticosteroid treatment was discontinued, and they were prescribed methylprednisolone tablets at a 4mg dose to be taken orally. Additionally, they were instructed to take azithromycin tablets at a 0.25mg dose twice a day and aluminum magnesium carbonate chewable tablets at a 0.5g dose three times a day. Despite experiencing repeated psychiatric symptoms, the patient was discharged from the hospital without experiencing any seizures. Following discharge, the patient's steroid and antibiotic treatments were gradually phased out. We advised the patient's family to take the patient to a psychiatric hospital for further diagnosis and treatment of psychiatric symptoms, but unfortunately, the patient's family refused.

During the two-month follow-up, the patient was not taking anticonvulsant drugs and did not experience any seizures. Additionally, the rash had completely disappeared while the hyperpigmentation remained unchanged. The psychiatric symptoms observed in the patient were consistent with those experienced in the past. However, routine blood tests, including CRP, SAA, liver and kidney function, urine routine, coagulation routine, and D-dimer did not show any significant abnormalities.

**Figure 4:**
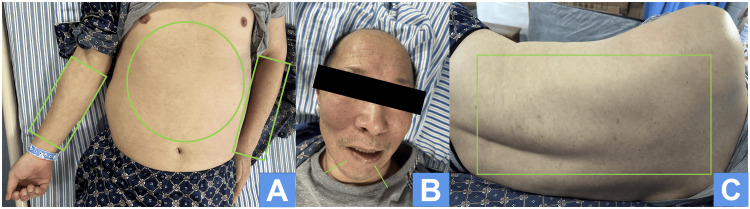
The rash of skin has almost completely disappeared on August 30, 2023 The rash on the abdomen and both upper extremities, represented by circles and rectangles (A), has completely vanished. Likewise, the mucous membrane ulceration and bleeding at the corners of the mouth, indicated by arrows (B), have completely resolved. Additionally, the rash on the back, depicted as rectangle (C), has almost completely disappeared.

On August 30, 2023 (nearly 19 months after the onset), we approached the patient, who was undergoing physical rehabilitation at a local rehabilitation hospital, to inquire about the rash (Figures 4A-4C). According to his wife, the rash was not flaring up, although there was occasional reddening of the skin. The patient had stopped taking anticonvulsant medication since his discharge from the hospital and had not experienced any seizures. However, he was currently experiencing poor sleep, emotional instability, constant worry about the recurrence of his rash, feelings of depression and sadness, and always suspecting infidelity by his wife and demanding that he keep her by his side at all times. To aid his sleep, he was now only taking estazolam tablets 1 mg.

For the convenience of the readers, we present a table of the manifestations of the rash over time (Table 1). 

**Table 1:** The manifestations of the rash over time

Date	Rash Presentation
2022/2/4 (2 days after the onset)	Pale red macules appeared on chest and back, some fused into small patches.
	No skin tenderness, no ulceration.
2022/2/6 (4 days after the onset)	The rash on the chest and back turns dark red and blends into patches.
	Blood-red colored maculopapular rash appears on the inner side of both upper limbs and extends to its outer side.
	The skin was slightly indurated without ulceration.
2022/2/8 (6 days after the onset)	The dark red rash on the chest and back blends into a large area.
	Soybean-sized blisters appear on the inside of both upper limbs.
	Dark red rash occurred on both lower limbs.
	Dark red petechiae appeared between the base of the tail and the buttocks.
	Skin tenderness, pronounced at night.
2022/2/11 (9 days after the onset)	Puffiness of head and face, congestion of lids and mucous membranes, rash on chest and back fused into pieces, partially broken, pus oozing out.
	The blisters on the inner side of both upper limbs were not increased, the surrounding rash turned dark and fused into small patches.
	The rash on both lower limbs increased and did not ulcerate.
	Skin slightly ulcerated at scrotum and penis.
	Scattered large blisters on the trunk with up to 80% of the skin lesions and intense tenderness.
2022/2/15 (13 days after the onset)	Ulceration of the mucous membrane of the mouth, ulceration and bleeding of the mucous membrane of the corners of the mouth.
	The mucous membrane of the eyelids is broken.
	Rash involving chest, neck, shoulders and back, fused into a large area, partly ulcerated with oozing.
	Most of the rash on the limbs ulcerated, rash not added.
	The blisters in the axillae and coccyx increased and partly ulcerated.
	Scrotum enlarged, with herpes around the penis.
	Tenderness is intense.
2022/2/21 (19 days after the onset)	The prior appearance of the rash gradually subsided.
	The blisters on the chest, back and inner limbs ulcerate and ooze diminishingly.
	Swelling of the head and face gradually disappears.
	Exudation from the damaged mucous membranes of the mouth and eyelids decreases.
	Retraction of the scrotum and reduction of penile herpes.
	Tenderness decreases.
2022/2/27 (25 days after the onset)	The rash basically subsides, a large amount of dark brown dead skin flaked off, and the broken epidermis dries and crusts.
	The broken mucous membranes of the oral cavity, corners of the mouth and eyelids were basically restored.
	Tenderness basically disappeared.
2022/3/3 (29 days after the onset)	The rash was largely resolved.
	A few hyperpigmented spots remain on the inner limbs.
Within 2 months of discharge	The rash disappears completely.
	A few hyperpigmented spots remain.
2023/8/30 (nearly 19 months after the onset)	Occasional redness of the skin.
	No obvious hyperpigmentation.

## Discussion

Lamotrigine is a recently developed aromatic anticonvulsant drug that is currently the preferred choice for treating partial and generalized seizures due to its superior efficacy [4]. However, it is important to note that there are potential adverse reactions associated with its use, such as rash, dizziness, nausea, diplopia, and ataxia [5]. One of the most frequent adverse events, occurring in approximately 10% of cases, is rash. This skin rash can take on various forms and, in severe cases, can be life-threatening, as in SJS and TEN [3]. The incidence of rash is positively correlated with both the initial dose and the incremental rate of lamotrigine [6]. The more the initial dose, the higher the rate of skin rash. In a study [7] of 3,071 patients who used lamotrigine, Werz found that the rate of skin rash was 1% with an initial dosage of 25 mg, 9% with an initial dosage of 50 mg, 12% with an initial dosage of 100 mg, and 38% with an initial dosage of 200 mg (Table 2).

**Table 2:** Correlation between initial dose of lamotrigine and rate of skin rash

initial dose (mg)	rate of skin rash(%)
25	1
50	9
100	12
200	38

For adults and children over 12 years of age, it is recommended to start with an initial dose of 25 mg every other day for two weeks. This should be followed by a 25 mg dose once daily for the next two weeks. After that, the dosage can be increased by 25 mg to 50 mg every one to two weeks, with a maintenance dose of 100 mg to 200 mg per day typically prescribed [8]. According to research [4], the incidence of rash is only 1.5% when the dosage of sodium valproate is increased to 62.5 mg in the fifth week. However, the incidence of rash increases to 12% even when the dosage is increased to 375 mg. It is worth noting that sodium valproate is a traditional non-aromatic anticonvulsant drug that rarely causes severe rash when used alone [9]. Sodium valproate, due to its zoological properties as a CYP isoenzyme inhibitor, increases the serum concentration of lamotrigine and its metabolites when used [10]. Meanwhile, due to competition between the two drugs for the common ametabolous pathway of intrahepatic glucuronide [11] there is more than doubling the elimination half-life of lamotrigine. Because of the correlation between lamotrigine serum levels and the seizure symptom, the combination of sodium valproate and lamotrigine is often used in clinical treatment for refractory epilepsy [12]. However, exceeding the recommended initial dose or incremental rate of lamotrigine can increase the incidence of fatal rash. It is important to note that frequent drug adjustments are also positively associated with the incidence of the rash [13]. Apart from pharmacological interventions, the occurrence of rash can also be associated with human leukocyte antigen (HLA). In the Chinese population, the presence of HLA-B*15:02 and HLA-A*24:02 alleles have been found to induce SJS/TEN, while the latter allele is also associated with typhus and papule. Conversely, the HLA-B*33:03 allele is known to be a protective gene against SJS/TEN [14]. Furthermore, the combination may lead to the manifestation of psychiatric symptoms that could be linked to the phenomenon of “forced normalization” resulting from elevated levels of lamotrigine in the bloodstream [15].

TEN is a type of hypersensitivity reaction that can be triggered by certain drugs. It is primarily characterized by skin and mucosal damage, including erythema, hemorrhagic erosions, blistering, necrotic epidermal peeling, and skin nudity. If lesions cover more than 30% of the body surface area, it is diagnosed as TEN. If lesions cover less than 10% of the body surface area, it is called SJS [16]. Standard clinical treatment for epilepsy involves anticonvulsant drug single therapy, which successfully relieves seizures in approximately 70% of patients. However, the remaining 30% of patients may require combination drug therapy to manage their seizures effectively [2]. In this case, the use of lamotrigine alone was not effective in controlling the symptomatic epilepsy secondary to cerebral hemorrhage. Nevertheless, the combination of sodium valproate showed positive results in controlling seizure symptoms. Unfortunately, after about two weeks of taking the combination, the patient developed a rash that gradually worsened. In severe cases, the rash manifested as dark purple blisters on the trunk, neck, face, and upper extremities, mucosal erosion and ulceration of the cheeks, eyes, mouth, and peri-genital area, large epidermal necrosis, and desquamation, leaving exposed skin. The lesions covered more than 80% of the patient's body. The patient had never experienced an allergic reaction to seafood since childhood, therefore, food-related factors were ruled out. Additionally, there was no history of exposure to any potential allergens or known allergies. Prior to the onset of the TEN, the patient had been taking lamotrigine, sodium valproate, and a calcium supplement regularly. The patient had been taking the calcium supplement for several years without experiencing any discomfort or adverse reactions related to the rash. According to a study [17], the probability of severe rash in adults taking lamotrigine independently is 0.1%. Most rashes occur within 14 days [18], with half occurring within 19.5 days. Valproate-induced rash is mostly triggered after a year of drug use [19]. While there is no reliable evidence that valproate can cause TEN on its own, SJS/TEN has been reported in the literature when certain anticonvulsant drugs, such as carbamazepine [20] and lamotrigine [21], are combined with valproate. The case of TEN developed after 30 days of administration of lamotrigine, based on our analysis, we believe that this was caused by a combination of lamotrigine and sodium valproate. Based on the metabolic mechanism of both drugs [1], it appears that sodium valproate enhances the ability of lamotrigine to induce TEN. In a study by Page et al. [21], a 54-year-old American man with glioblastoma multiforme brain tumor and complex partial epilepsy was prescribed valproate up to 1,500 mg within one week. The dose of lamotrigine was also increased to 100 mg/day within one week. Unfortunately, the patient died after 17 days of treatment due to an outbreak of TEN. The autopsy report suggests that the cause of death may have been due to the rapid increase of lamotrigine dosage within a week, instead of the recommended two weeks, and the concomitant administration of sodium valproate, which led to elevated serum lamotrigine levels. In a case report by Lu [22], a patient with epilepsy secondary to cerebrovascular disease was treated with sodium valproate 500 mg and lamotrigine. The initial dose of lamotrigine was 25 mg once a day, which was then increased to 25 mg twice a day after three days. After 17 days, the dose was further increased to 25 mg in the morning and 50 mg in the evening. The patient was diagnosed with TEN 20 days later and was treated symptomatically and supportively for a month, resulting in improvement. Both studies demonstrated that TEN occurred following the use of combined medication and rapid dose increase.

TEN is a highly dangerous disease, with an incidence of only 1.9 cases per million inhabitants per annum. Its overall mortality rate is 15% and can range from 25% to 35% for skin peeling areas greater than 30%, and up to 50% in elderly patients [23]. The survivors of TEN may experience long-term effects such as permanent mucosal damage, skin pigmentation, and scarring. Additionally, they may suffer from psychiatric and psychological symptoms, which are often overlooked in clinical practice despite being more distressing for the patients [24]. A study conducted on the psychological impact of SJS/TEN on patients [25] found that post-traumatic stress disorder (PTSD), anxiety, and depression were among the most devastating consequences. Patients frequently experience inadvertent flashbacks, which can cause them to live in constant fear of a relapse. The occurrence of these sequelae is quite high as evidenced by a study conducted by Dodiuk-Gad et al. [26]. The study revealed that 71% of patients experienced significant emotional distress while 29% may have PTSD. Additionally, 65% of patients displayed post-traumatic stress symptoms, which is a major long-term complication of SJS/TEN. This complication is often observed in psychologically vulnerable patients [27]. This patient exhibited sleep disturbance, anxiety, and depression, including fear of rash recurrence, suspicious hallucinatory symptoms that the patient saw a recently deceased patient from another ward visited the patient's bedside at noon, described the encounter in great detail, and delusional symptoms that the patient expressed suspicions of infidelity by his wife after she had a brief conversation with other men. Additionally, hyperpigmentation was observed on the skin after it had healed.

The basic treatment principle for acute stage SJS/TEN is supportive therapy, as it is a delayed metaplasia. According to the 2021 Delphi International, Multidisciplinary Consensus [28], timely discontinuation of medication is recommended as a specific action. This paragraph outlines the necessary steps for ensuring patient safety and comfort during medical care. These steps include maintaining vital signs, assessing and relieving pain, keeping the room temperature between 25°C to 32°C, preventing infection by maintaining a clean environment, and practicing proper hand hygiene when interacting with the patient. Additionally, infection indicators should be monitored and antibiotics used if necessary. In order to treat damaged skin, it is important to protect it and aspirate any blisters that may have formed. However, it is not recommended to actively peel off any necrotic herpetic skin. To cover all damaged skin, including bare skin, it is best to use non-adherent exciple. It is also important to control any inflammatory exudate and adhesions that may occur in the oral or genital mucosa. Providing adequate enteral and/or intravenous nutritional support is crucial, as well as assessing and intervening in any psychiatric symptoms that may arise. Thrombosis should be prevented, as well as stress ulcers. If necessary, ICU intervention may be required. High-dose glucocorticoid shock therapy is frequently utilized as the preferred clinical treatment [29] and has been shown to have a favorable impact on severe conjunctivitis associated with TEN [30].

Watanabe et al. proposed nine risk factors associated with poor prognosis based on the internationally customary TEN disease severity scoring system (SCORTEN) [31]. These risk factors include persistent rapid ventricular rate above 120 beats/min; time between the discovery of the rash and the start of treatment > 8 d; serum bicarbonate concentration< 20 mmol/L; serum glucose level > 14 mmol/L. respiratory disease within two days after the discovery of the rash; epidermal peeling area > 10% of the body surface area; the presence of the malignant tumor; serum urea nitrogen > 28 mg/mL; age between 40 and 71 years. In addition to the aforementioned therapeutic measures, early administration of intravenous immunoglobulin infusion and/or plasma exchange is necessary to reduce morbidity and mortality rates in critical cases with poor prognosis [31]. According to studies [32], TNF-α inhibitors such as etanercept have been found to be more effective in repairing skin and less damaging to the gastrointestinal tract compared to glucocorticoids. The patient's treatment aligns with these principles. To prevent severe liver damage, it is recommended to take precautionary measures to protect the liver since it is the primary site for the metabolism of lamotrigine. Maintaining a stable liver function is crucial for effective treatment and prognosis.

## Conclusions

This case suggests the following recommendations: It is preferable to use the drug alone, if possible. If sodium valproate needs to be combined with lamotrigine, the initial dose should be low and slowly increased. Prior to treatment, a detailed history of drug and food allergies should be obtained. If available, genetic testing should be performed, and blood drug concentrations and liver function should be monitored regularly during treatment. The rash typically appears within the original eight weeks, with a higher likelihood in the first two weeks. Early detection and treatment are crucial in preventing progression towards SJS/TEN. If SJS/TEN does occur, appropriate glucocorticoid shock therapy, intravenous infusion of corticosteroid immunoglobulin, preemptive liver-protective therapy, and maintaining stable liver function are key components of treatment and can have a positive effect on prognosis. In order to provide comprehensive care, patients must be monitored for any psychiatric symptoms resulting from the possibility of a severe rash on anticonvulsant medications. As with other symptomatic psychiatric disorders that occur after severe physical injury, SJS/TEN can result in persistent psychiatric symptoms such as PTSD, anxiety, depression, sleep disorders, and hallucinatory delusions. These symptoms are often initially overlooked by clinicians until the symptoms worsen significantly. Therefore, it is important for clinicians to consciously inquire about the mental status of SJS/TEN patients who have not yet developed significant psychiatric symptoms. By detecting minor psychiatric symptoms early, various therapies including medication, physiotherapy, and psychotherapy can be used as early interventions to prevent the deterioration of psychiatric symptoms to an unmanageable level.
